# GEOGRAPHIC DISTRIBUTION OF THE CERTIFIED NURSING ASSISTANT WORKFORCE IN U.S. COUNTIES

**DOI:** 10.1093/geroni/igae098.3990

**Published:** 2024-12-31

**Authors:** Mallory Johnson

**Affiliations:** University of California Davis, Davis, California, United States

## Abstract

Although geographic disparities in LTSS access are well documented, no studies exam the spatial variation in the workforce primarily responsible for delivering direct care. This study assesses the distribution of the certified nursing assistants (CNA) and the community characteristics associated with CNA employment. A cross-sectional design is used to explore the relationship between CNA employment and health care delivery and training infrastructure, local labor market structure, and population characteristics. Data from CMS’s Payroll Based Journal, the American Community Survey, and the National Conference of State Legislatures are fitted to a negative binomial hurdle model. 2,871 counties employ 1,616,798 CNAs (“count locations”). Only 271 counties employ no CNAs (“zero locations”). Zero locations are more likely to be rural and have lower population, less hospitals, and fewer CNA training programs even though their aging population is higher. Regression results demonstrate that the number of hospitals, nursing home beds, and total population are positively associated with CNA employment in both count and zeros locations. Medicaid expansion, the foreign-born population, and the proportion of the workforce employed in hospitality or public sector jobs, and land area are negatively associated with CNA employment in both count and zero locations. Differential effects are seen for the number of CNA training programs, population with a disability, and insurance status. CNA employment is highly variable. These findings confirm the “inverse care law” by demonstrating that CNA employment is not associated primarily with population need but with other health care, labor market, and population factors.

